# NEREL-BIO: a dataset of biomedical abstracts annotated with nested named entities

**DOI:** 10.1093/bioinformatics/btad161

**Published:** 2023-04-02

**Authors:** Natalia Loukachevitch, Suresh Manandhar, Elina Baral, Igor Rozhkov, Pavel Braslavski, Vladimir Ivanov, Tatiana Batura, Elena Tutubalina

**Affiliations:** Lomonosov Moscow State University, Moscow 19899, Russia; Madan Bhandari University of Science and Technology, Chitlang 44600, Nepal; Madan Bhandari University of Science and Technology, Chitlang 44600, Nepal; Lomonosov Moscow State University, Moscow 19899, Russia; Ural Federal University, Yekaterinburg 620002, Russia; HSE University, Moscow 101000, Russia; Innopolis University, Innopolis 420500, Russia; A.P. Ershov Institute of Informatics Systems, Novosibirsk 630090, Russia; HSE University, Moscow 101000, Russia; Artificial Intelligence Research Institute, Moscow 105064, Russia; Sber AI, Moscow 121170, Russia

## Abstract

**Motivation:**

This article describes NEREL-BIO—an annotation scheme and corpus of PubMed abstracts in Russian and smaller number of abstracts in English. NEREL-BIO extends the general domain dataset NEREL by introducing domain-specific entity types. NEREL-BIO annotation scheme covers both general and biomedical domains making it suitable for domain transfer experiments. NEREL-BIO provides annotation for nested named entities as an extension of the scheme employed for NEREL. Nested named entities may cross entity boundaries to connect to shorter entities nested within longer entities, making them harder to detect.

**Results:**

NEREL-BIO contains annotations for 700+ Russian and 100+ English abstracts. All English PubMed annotations have corresponding Russian counterparts. Thus, NEREL-BIO comprises the following specific features: annotation of nested named entities, it can be used as a benchmark for cross-domain (NEREL → NEREL-BIO) and cross-language (English → Russian) transfer. We experiment with both transformer-based sequence models and machine reading comprehension models and report their results.

**Availability and implementation:**

The dataset and annotation guidelines are freely available at https://github.com/nerel-ds/NEREL-BIO.

## 1 Introduction

The lack of richly annotated training datasets is a well-known challenge in developing biomedical entity extraction systems. Despite having a large number of resources in the general domain, many languages have not made significant progress in the biomedical field. Russian is one such example; it is one of the top 10 languages in the world and has many natural language processing (NLP) datasets and resources, but the biomedical part of Russian NLP is underdeveloped. In particular, the Russian part of the Unified Medical Language System (UMLS) (Bodenreider 2004) includes three source vocabularies, and it still only amounts to 1.8% of the English UMLS in vocabulary and 1.36% in source counts (NIH UMLS 2022). Currently, there are several annotated corpora for the extraction of diseases, drugs, and adverse drug reactions from social media and clinical records in Russian (Tutubalina et al. 2021; Nesterov et al. 2022). A recent work on a Russian medical language understanding benchmark (Blinov et al. 2022) includes the RuDReC corpus (Tutubalina et al. 2021) for named entity recognition (NER). However, these corpora do not cover scientific texts and include flat (non-nesting) entity mentions only.

The majority of existing datasets and NER methods have been designed for capturing flat (non-nesting) mention structures over coarse entity type schemes. Moreover, the annotated entities in these corpora are limited to the most common entity types such as drugs/chemicals and diseases (Leaman et al. 2009; Gurulingappa et al. 2010; Van Mulligen et al. 2012; Wei et al. 2016); see an overview of 20+ NER biomedical datasets in a BIGBIO (BigScience Biomedical) library (Fries et al. 2022) for more information. Recent work has shown an increased interest in nested entity structures in general-domain data in various languages, including English (Ringland et al. 2019), Russian (Loukachevitch et al. 2021), Thai (Buaphet et al. 2022), and Danish (Plank et al. 2020). The most widely studied corpus for nested NER in the biomedical domain is GENIA (Kim et al. 2003) which consists of 2000 PubMed abstracts and 100 000 annotations divided into 47 entity types. Yet, only 17% of the entities in the GENIA corpus are nested within another entity (Katiyar and Cardie 2018). An another large concept-mention annotated dataset named MedMentions (Mohan and Li 2018) contains 4392 PubMed abstracts annotated with 21 entity types, including disorders, anatomical structures, chemicals, and also some general concepts such as organizations, population groups, etc. However, the authors chose to annotate the most specific concept in texts without any overlaps in mentions.

**Figure 1:**
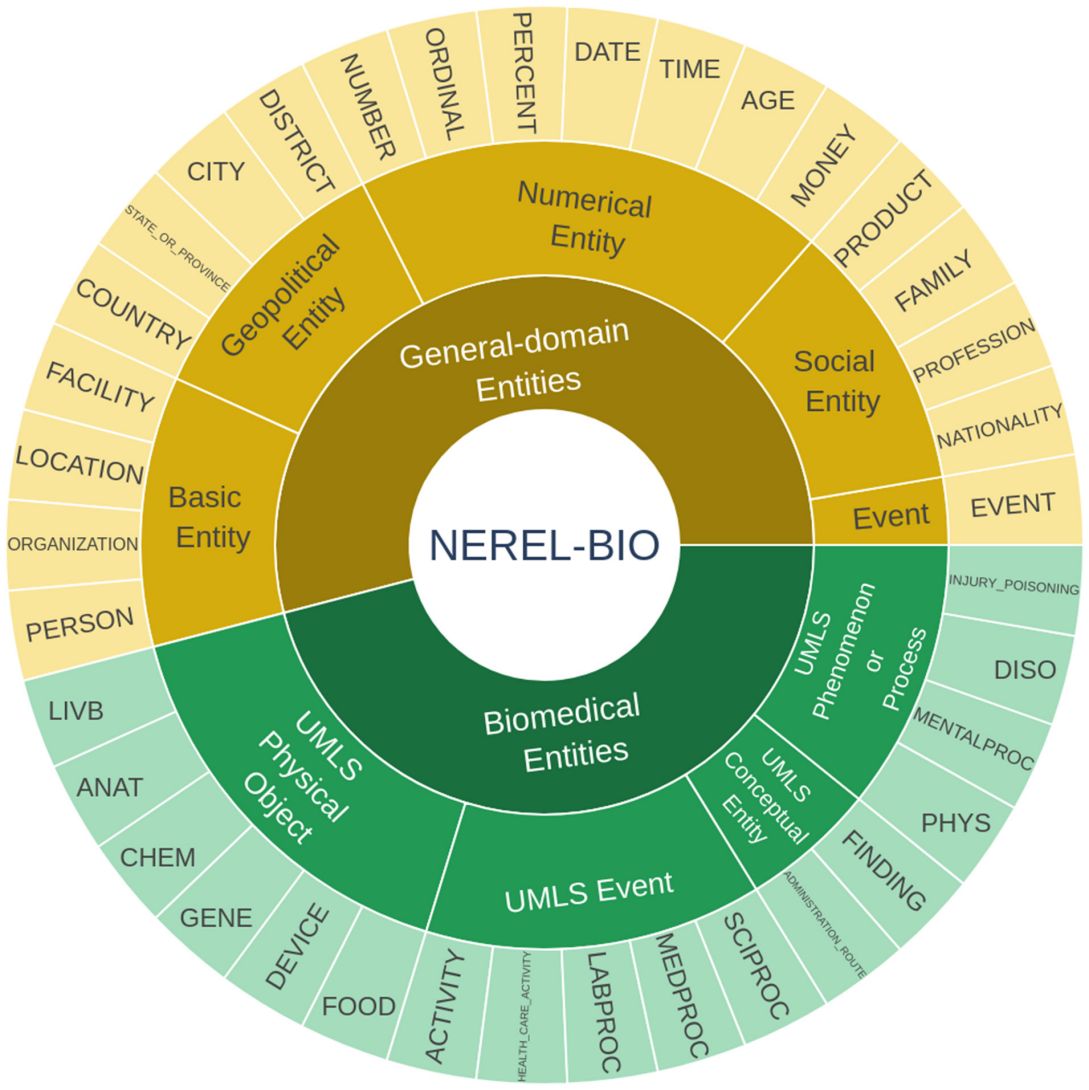
An overview of named entity types in NEREL-BIO corpus. Our corpus contains 20 and 17 fine-grained general-domain and biomedical entity types, respectively

**Figure 2:**
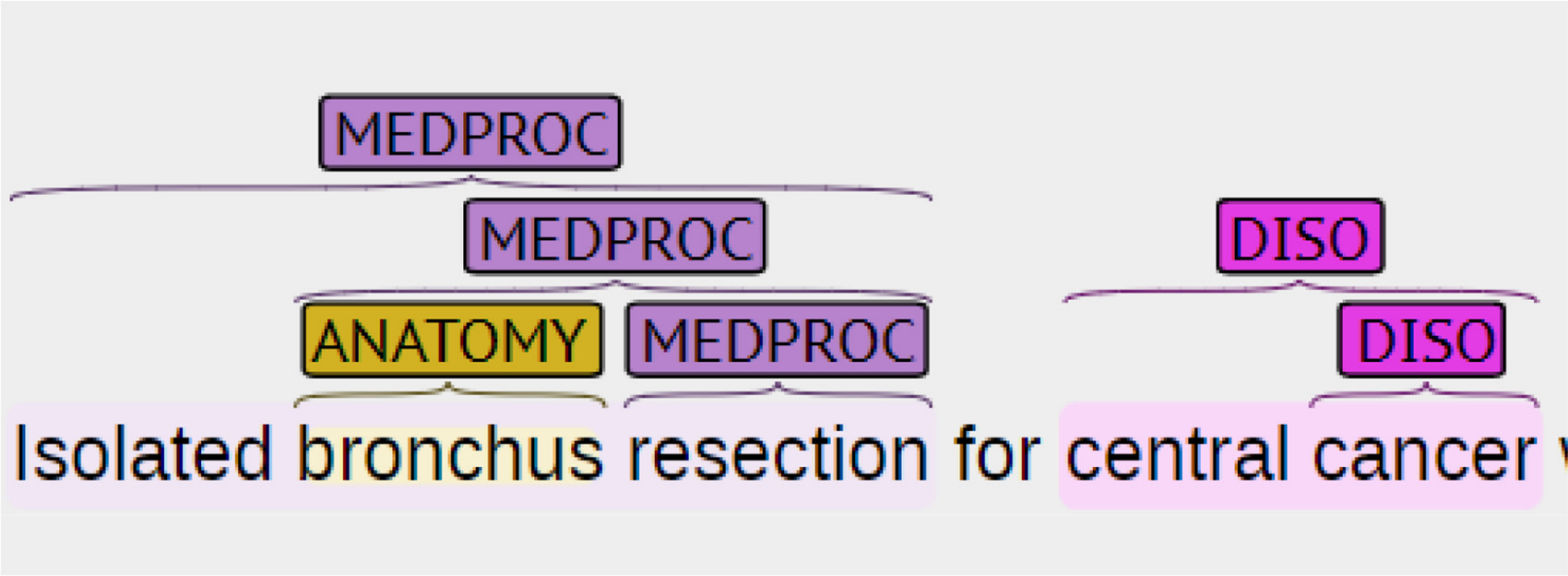
Example of nested named entities in NEREL-BIO. Internal entities “bronchus”, “bronchus resection”, “resection” are available in UMLS. Longer entities “isolated bronchus resection” and “central cancer” are important for establishing appropriate relations

To encourage the development of state-of-the-art information extraction systems aimed at providing more comprehensive coverage of biomedical concepts, we decided to construct a large nested named entity dataset NEREL-BIO over Russian PubMed abstracts. All entity mentions, including nested structures with up to six layers of depth, are manually annotated. In addition to the nested entity structure, we have more 30+ entity types from both general-domain and biomedical fields, as shown in Fig. 1. Figure 2 presents an example of nested named entities in NEREL-BIO. The source abstract discusses “isolated bronchus resection for central cancer” and provides the results of surgical treatment in these specific conditions. Entities “bronchus”, “bronchus resection”, “resection” are included in UMLS, while “isolated bronchus resection” and “central cancer” are not. Nested entity annotations create a basis for establishing relations between correct (longer) entities, as well as linking internal entities to equivalent UMLS concepts.

The main contributions of our work can be summarized as follows:

1. We present NEREL-BIO, a new biomedical dataset for nested NER in Russian accompanied with a smaller English corpus.
2. We evaluate BERT-based machine reading comprehension (MRC) and sequence models for biomedical nested NER.
3. To promote further cross-lingual research, we annotate a subset of 100+ English abstracts in translation from Russian using the same annotation scheme.

## 2 Data collection and annotation

NEREL-BIO extends the annotation scheme of the general-domain Russian dataset NEREL (Loukachevitch et al. 2021) annotated with nested named entities. Since NEREL-BIO includes a significant part of NEREL entity types, we provide a short description of NEREL entity types in Section 2.1. In Section 2.2.2, we describe 17 biomedical entity types that have been utilized in NEREL-BIO (including DISO entity, which is renamed DISEASE from the general NEREL dataset).

### 2.1 Overview of NEREL nested named entities

NEREL is the first Russian dataset annotated simultaneously with nested entities, relations between those entities, and knowledge base links (Loukachevitch et al. 2021, 2022). Entity linking annotations leverage nested named entities, and each nested named entity can be linked to a separate Wikidata entity. Nested entities enhance coverage of annotated entities, as well as relations between entities and knowledge base links. For example, using single ORGANIZATION entity in *Lomonosov Moscow State University* leads to the loss of two internal named entities: CITY (*Moscow*) and PERSON (*Lomonosov*). In such cases, NEREL employs the nested annotation *Lomonosov*${}_{\mathrm{PERSON}}$*Moscow*${}_{\mathrm{CITY}}$*State University*. Annotation of internal entities allows “local” relations between nested entities. In the above example, it allows for establishing a relation between the university and its headquarters (*Moscow*). Annotated nested entities enable wider coverage of entity linking into a knowledge base. For example, entity *Mayor of Novosibirsk* is absent in Wikidata (which is a reference knowledge base for NEREL), but nesting permits linking of the internal entities *Mayor* and *Novosibirsk*.

At the time of this writing, NEREL contains 56K named entities and 39K relations annotated in 900+ person-oriented news articles. Twenty-nine entity types in NEREL can be categorized as follows:

- basic entity types: PERSON, ORGANIZATION, LOCATION, FACILITY;
- geopolitical entities subdivided into COUNTRY, CITY, DISTRICT, and STATE_OR_PROVINCE entities;
- numerical entities: NUMBER, ORDINAL, DATE, TIME, PERCENT, MONEY, AGE;
- socio-political entities: NATIONALITY, RELIGION, IDEOLOGY, FAMILY, LANGUAGE;
- law-related entities: LAW, CRIME, PENALTY;
- work-related entities: PROFESSION, WORK_OF_ART, PRODUCT, AWARD;
- DISEASE for labeling various health disorders and symptoms;
- EVENT, which is used for labeling so called “news events” as opposed to everyday or regular activity.

We considered the NEREL entity types for inclusion into NEREL-BIO to be able to describe the most important social relations of biomedical entities. The DISEASE entity type is most relevant to the biomedical domain. Also it was decided to use in the NEREL-BIO labeling the following NEREL general entity types: eight basic entity types, seven numerical entities, tags for characterizing persons (NATIONALITY, PROFESSION, and FAMILY), PRODUCT and EVENT. EVENT entity is used for labeling such situations as epidemics, military conflicts, tsunamis, etc., mentioned in connection with the spread of diseases or the need for additional medical care. The EVENT entity mainly corresponds to Environmental event and Traumatic event concepts in UMLS. In total, 20 general-domain entity types are available for annotating biomedical texts.

It can be also noted that PERSON, ORGANIZATION, and LOCATION entities from the basic entity group in the general domain were annotated in the biomedical MedMentions corpus (Mohan and Li 2018), locations were also annotated in the QUAERO corpus (Névéol et al. 2014). The NEREL PROFESSION type corresponds to the MedMentions occupation (OCCU) type.

### 2.2 NEREL-BIO dataset

#### 2.2.1 Text collection

We used sourced documents from the WMT-2020 Biomedical Translation Task collection (Bawden et al. 2020) that contains 6029 Medline abstracts in Russian and their English translations (https://github.com/biomedical-translation-corpora/corpora). We selected texts in the range of 6–20 sentences. We trained a mBERT-based (Devlin et al. 2019) NER model on MedMentions (Mohan and Li 2018) and applied it to Russian abstracts in a zero-shot fashion. We picked about 100 documents with the densest and most diverse recognized entities. Based on the analysis of this automatic annotation, we selected abstracts with disease mentions and related laboratory or medical procedures for including in NEREL-BIO.

The abstracts were annotated using the BRAT annotation tool (Stenetorp et al. 2012). To facilitate manual annotation, automatic preannotation was done with two models. First, multilingual BERT (Devlin et al. 2019) trained on the English MedMentions (Mohan and Li 2018) was applied for biomedical entity recognition: 10 biomedical entity types corresponding to UMLS semantic types [https://lhncbc.nlm.nih.gov/semanticnetwork/download/SemGroups.txt. The following semantic tags were used in pre-annotation: ACTI (activities), ANAT, CHEM, DEVI (devices), DISO, LIVB, ORGA (organizations), PHEN (Phenomena), PHYS (Physiology), and PROC (procedures)]. The second model used in automatic preannotation was MRC model (Li et al. 2020) trained on the NEREL dataset, that helped labeling nested entities from the general domain. The automatic techniques provided the annotation of most evident entities and became a basis for further manual labeling. The preannotated abstracts were manually annotated by experts with further control by the moderator. We also annotated 105 English abstracts which are translations of initially selected Russian abstracts for future cross-lingual studies and experiments.

Table 1 summarizes statistics of NEREL-BIO in terms of documents and entity mentions. Table 2 contains most frequent disease mentions in the Russian part of NEREL-BIO. It can be seen that the selected abstracts are quite diverse in content.

**Table 1:** Statistics of NEREL-BIO.

Collection	No. of doc	No. of entities	No. of nonzero entity types
Abstracts in Russian	766	66 888	37
Abstracts in English	105	10 651	32

**Table 2:** Ten most frequent disorders (diseases and symptoms) in NEREL-BIO (translated from Russian).

Disease	No. of mentions
Tumor	198
Diabetes	112
Pain	111
Cancer	101
Tuberculosis	100
Arterial hypertension	88
Infection	86
Stroke	85
Cardiac ischemia	82
Alzheimer’s disease	69

#### 2.2.2 Entity types

Biomedical entity types selected for annotation are based on their presence in the UMLS taxonomy and other annotated datasets in the biomedical domain. Seventeen specialized biomedical entity types and 20 entity types from the general NEREL dataset are included into the NEREL-BIO annotation scheme. Table 3 presents linking of the NEREL-BIO entity types to UMLS semantic types and most relevant concepts. Table 3 also contains entity statistics for the Russian and English parts of the NEREL-BIO corpus. The full set of entity types, explanations, and examples in NEREL-BIO are presented in Table 4.

**Table 3:** UMLS semantic groups and NEREL-BIO entity types.

UMLS semantic types	NEREL-BIO types	UMLS CUI	Initial dataset	Freq (Rus)	Freq (Eng)
A1 Physical object					
A1.1 Organism	LIVB	C0029235	NEREL-BIO	730	69
A1.1.3.1.1.4.1 Human	PERSON	C0086418	NEREL	7258	907
A1.2 Anatomical structure	ANATOMY	C1268086	NEREL-BIO	8992	1842
A1.2.3.5 Gene or Genome	GENE	C0008633	NEREL-BIO	376	2
A1.3 Manufactured Object					
	MONEY	C087090	NEREL	35	0
A1.3.1 Medical device	DEVICE	C0025080	NEREL-BIO	661	40
	PRODUCT	C1514468	NEREL	85	4
A1.4.1 Chemical	CHEM	C0220806	NEREL-BIO	5049	1109
A1.4.3 Food	FOOD	C0016452	NEREL-BIO	135	10
A2 Conceptual entity					
A2.1 Idea or Concept					
A2.1.1 Temporal concept	DATE	C0011008	NEREL	1410	217
	TIME	C0040223	NEREL	128	40
A2.1.3 Quantitative concept	NUMBER	C0237753	NEREL	5202	724
	PERCENT	C0439165	NEREL	1948	175
	AGE	C0001779	NEREL	505	59
A2.1.4 Functional concept	ADMINISTRATION_ROUTE	C0013153	NEREL-BIO	295	58
A2.1.5.4 Geographical areas	LOCATION	C0450429	NEREL	166	0
	COUNTRY	C0454664	NEREL	473	20
	STATE_OR_PROVINCE	C1555317	NEREL	81	0
	DISTRICT	C5447118	NEREL	17	0
	CITY	C0600182	NEREL	104	11
	FACILITY	C1547538	NEREL	214	36
A2.2 Finding	FINDING	C2825141	NEREL-BIO	2965	709
A2.4 Intellectual product	ORDINAL	C0439080	NEREL	1203	61
A2.7 Organizations	ORGANIZATION	C1561598	NEREL	531	18
A2.8 Group attribute	NATIONALITY	C0027473	NEREL	25	1
A2.9 Group					
A2.9.1 Professional group	PROFESSION	C1527116	NEREL	237	22
A2.9.3 Family group	FAMILY	C0015576	NEREL	30	0
B Event	EVENT	C0456156, C4751223	NEREL	43	4
B1 Activity					
B1.1.2 Individual behaviour	ACTIVITY	C0004927	NEREL-BIO	424	32
B1.3.1 Health care activity	HEALTH_CARE_ACTIVITY	C0086388	NEREL-BIO	394	28
B1.3.1.1 Laboratory procedure	LABPROC	C0679541	NEREL-BIO	1644	308
B1.3.1.3 Therapy or preventive procedure	MEDPROC	C0087111	NEREL-BIO	4204	458
B1.3.2 Research activity	SCIPROC	C0242481	NEREL-BIO	1835	519
B2 Phenomenon or process					
B2.2.1.1 Physiologic function	PHYS	C0031845	NEREL-BIO	6769	973
B2.2.1.1.1.1 Mental process	MENTALPROC	C0025361	NEREL-BIO	443	65
B2.2.1.2 Pathologic function	DISO	C0677042	NEREL	11 576	2020
B2.3 Injury and poisoning	INJURY_POISONING	C0178314	NEREL-BIO	683	115

**Table 4:** NEREL-BIO entities and their explanations.

NEREL-BIO entity	Explanation	Examples in the dataset (translated from Russian)
ACTIVITY	Human or animal behavior	Smoking, contact with animals, to give up smoking, military actions, and illegal drug use
ADMINISTRATION_ROUTE	Ways of administering a drug or another chemical to an organism for treatment including drug forms	Enteral, rectal, intranasal, intravenous, inhalation, injection, infusion
ANATOMY	Comprises organs, body part, cells and cell components, body substances	Eye, bone, brain, lower limb, oral cavity, blood, anterior lens capsule, right ventricle, lymphocyte
CHEM	Chemicals including legal and illegal drugs, biological molecules	Opioid, lipoprotein, iodine, adrenalin, memantine, methylprednisolone
DEVICE	Manufactured objects used for medical purposes	Catheter, prosthesis, tonometer, tomography removable prosthesis, stent, metal stent
DISO	Any deviations from normal state of organism: diseases, symptoms, dysfunctions, abnormality of organ, excluding injuries or poisoning	Appendicitis, hemorrhoids, magnesium deficiency deep vein thrombosis, diabetes mellitus, pain spine pain, complication, bone cyst, acute inflammation
FINDING	Conveys the results of scientific study, experiments described in the abstract	Longer hospital stay, stopped the progression of keratoconus, stabilize the glaucoma process
FOOD	Substances taken in by the body to provide nourishment including fresh water, alcoholic beverages	Salt, milk, hot meal breast milk (in context of breastfeeding)
GENE	Nucleic acid sequences that function as units of heredity including DNA and chromosomes	KIR gene, PARK2, RASSF1A, allele, CYP2C19
HEALTH_CARE_ACTIVITY	Health care administration and organization activities	Hospitalization, discharge from hospital, medical evacuation
INJURY_POISONING	Damage inflicted on the body as the direct or indirect result of external force including poisoning	Overdosing, burn, drowning, falling, childhood trauma
LABPROC	Testing body substances and other diagnostic procedures such as ultrasonography	Biochemical analysis, polymerase chain reaction test electrocardiogram, histological
LIVB	Any individual living (or previously living) being except humans	Rat, mosquito, mouse, dog, parasite, rabbit, virus
MEDPROC	Procedures concerned with remedial treatment of diseases, including surgical procedures	Lung resection, antiviral therapy, eyelid surgery, chemotherapy, corneal transplant, appendectomy
MENTALPROC	Conceptual functions or thinking	Self-esteem, psycho-emotional status, cognitive function
PHYS	Biological function or process in organism including organism attribute (temperature) and excluding mental processes	Blood flow, childbirth, uterine contraction, arterial pressure, body temperature
SCIPROC	Scientific studies including mathematical methods or clinical studies, scales, classifiers, etc.	Retrospective analysis, confidence interval, ICD-10, analysis of variance, multivariate analysis

Biomedical entity types in NEREL-BIO are annotated according to UMLS definitions of relevant concepts. There are a few exceptions as given below:

- HEALTH_CARE_ACTIVITY, which is described as a quite general concept in UMLS, is treated as health care administration and organization activities such as *hospitalization* or *medical evacuation*;
- LABPROC entity comprises both laboratory and other diagnostic procedures;
- FINDING entity mostly corresponds to the *experimental finding* concept in UMLS and conveys the results of the scientific study presented in the abstract, e.g. *longer hospital stay, the progression of atherosclerosis*.

If compared with preannotation based on the MedMentions corpus, it should be noted that:

- from the LIVB entity, PERSON entity was singled out, because the annotated abstracts mainly discuss human diseases;
- PROC (procedures) were subdivided into scientific, medical, and laboratory procedures;
- from PHYS (physiology), the category of mental processes (MENTALPROC) was singled out;
- additional entities were annotated.

We calculated the intersection of the final annotation NEREL-BIO with initial automatic pre-annotation: the intersection contains about 25% spans with the same entity type, which means that the automatic preannotation was useful, but the annotation scheme was significantly changed.

It can be seen that all entity types of NEREL-BIO were successfully linked to the UMLS taxonomy. At the same time, we could see quite diverse mentions of geographical locations and some of the money (mainly in the context of medical expenses). Mentions of professions or occupations are quite frequent: mainly medical specialists are mentioned, but also there are studies on occupational diseases of specific professional groups.

Some principles of annotation employed in the general domain were changed in NEREL-BIO. In particular, in the general domain, mainly capitalized mentions were annotated as named entities. In the biomedical domain, the same entity types can also appear as lower-cased mentions:

- any humans or groups can be annotated with the label PERSON such as *patient*, *control group*, *population with low income*;
- ORGANIZATION tag is used not only for tagging specific organizations but organization types such as *hospital, medical institution, rehabilitation center*.
- location-related tags (LOCATION, COUNTRY, CITY, STATE_OR_PRO-VINCE, DISTRICT, FACILITY) are also used in both cases: *rural settlement, low-income countries, coastal areas, Brasilia, Vietnam*.

Entities annotated in NEREL-BIO can be absent in UMLS. For example, the term *left-sided congenital diaphragmatic hernia* is absent in UMLS. We annotate this as follows:


$$[\mathrm{left}-\mathrm{sided}\;[\mathrm{congenital}\;[{\left[\mathrm{diaphragmatic}\right]}_{\mathrm{ANATOMY}}{\left[\mathrm{hernia}\right]}_{\mathrm{DISO}}{]}_{\mathrm{DISO}}{]}_{\mathrm{DISO}}{]}_{\mathrm{DISO}}$$

Although we cannot link the whole term in UMLS, we can link the sub-terms: Hernia (C0019270), Diaphragmatic Hernia (C0019284), Respiratory Diaphragm (C0011980), and Congenital diaphragmatic hernia (C0235833).

For annotating multiword terms, we followed the following guidelines:

- up-to three–four word biomedical terms in form of noun groups without prepositions discussed in texts are annotated without additional checks;
- longer multiword phrases containing prepositions should be supported with some additional evidence, for example, there can be an abbreviation in the text for a long multiword term [*ST-segment elevation acute coronary syndrome* (*STSEACS*)], a long term or its English equivalent can be found in UMLS (*metastasis from malignant tumor of liver* C1282502) or other biomedical resources;
- internal spans in an annotated multiword term (single words or phrases), which can be considered to be valid biomedical terms, are also annotated with corresponding entity types;
- general adjectives, adjectival quantifiers are not included in the annotated entity: *various tumors* are annotated as $\mathrm{various}{\left[\mathrm{tumors}\right]}_{\mathrm{DISO}}$.

The annotation scheme was created during multi-round preliminary annotation of parallel Russian and English abstracts. Terminologists experienced in terminological studies including the biomedical domain were involved in the annotation. All annotated abstracts were additionally checked by a moderator.

In Table 5, we provide a brief summary of how frequently nested entities appear in NEREL-BIO. For each entity type, we counted how many times entities of this type appear as an outer entity (eliminating multiple occurrences of the same entity) and divide this number by the total occurrences of the entity type in the corpus. Then, we filter out the types with less than 200 occurrences in the corpus. The top 10 entity types along with their nestedness frequency are presented in the table. Frequencies in the parallel English/Russian abstracts of the NEREL-BIO are shown in the last two columns of Table 5. Here we compare only parallel abstracts for each language.

**Table 5:** Frequencies of top ten entity types with nested entities in full Russian collection and 100 Russian and English documents for comparison.

Entity type	**Full RU (%**)	**EN (%**)	**RU (%**)
FINDING	65.7	71.2	57.4
PHYS	38.3	40.7	39.8
INJURY_POISONING	37.7	49.0	39.4
DISO	37.3	41.2	37.6
DEVICE	33.9	42.5	46.2
LABPROC	30.2	34.8	31.0
MEDPROC	30.0	44.7	33.1
ANATOMY	27.3	28.3	31.0
SCIPROC	23.9	32.1	24.6
CHEM	22.5	20.1	17.2
Total entities	66 888	10 651	9209
Total (outer) nested entities	17 182	3002	2468

Frequencies of nested entities in Russian and the smaller English corpus are mostly comparable. The differences can be explained by the following: (i) the abstracts are not fully parallel: paper titles are absent in Russian abstracts but included in English abstracts; (ii) the different syntax of languages determines different structures of sentences and nestedness; (iii) sentences in Russian and in English are not always direct translations but can be significantly reformulated. Two last factors especially affected the FINDING entity since these can be long and therefore can be formulated in multiple ways.

Additionally, we analyzed nested entities in the following way. We aggregate typical pairs of nested entities from the corpus. Each pair has an outer and an internal entity. Table 6 presents top 10 pairs of types for such entities. Note, that an outer entity can contain one, two, or more internal entities. In fact, the NEREL-BIO dataset has outer entities that contain up to eight internal entities at the same level of nestedness.

**Table 6:** Top 10 nested entity pairs in NEREL-BIO.

Outer entity type	Internal entity type	Occurrences
DISO	DISO	3380
ANATOMY	ANATOMY	3051
DISO	ANATOMY	1476
PHYS	PHYS	1267
CHEM	CHEM	1116
PERSON	PERSON	1038
FINDING	PHYS	956
MEDPROC	MEDPROC	911
PHYS	ANATOMY	786
PHYS	CHEM	523
Total nested pairs		22 392

Therefore, we provide raw counts in the table. Overall, the Russian part of the NEREL-BIO contains 22 392 such pairs (the English subset has 3864 nested entity pairs).

To estimate the inter-annotator agreement (IAA) we calculate Krippendorff’s alpha (Krippendorff 2004). This coefficient measures data reliability by generalizing several known agreement indices and dealing with any number of categories, multiple scales (nominal, ordinal, binary, etc.), and incomplete or missing data (Checco et al. 2017). We performed calculations on a small piece of data (11 documents with about 850 entities) labeled by two independent annotators. We used an implementation from the software package krippendoff and obtained alpha of 0.823. This indicates good quality of agreement and reliability of annotations as noted by other researchers (Shabankhani et al. 2020; Campillos-Llanos et al. 2021).

## 3 Experiments and evaluation

For our experiments, we split NEREL-BIO into train/dev/test subsets (612/77/77 documents). For entity recognition experiments, we report results (see Table 1) on (i) Machine Reading Comprehension (MRC) model (Li et al. 2020) (Our code is available at https://github.com/fulstock/mrc_nested_ner_ru.) and (ii) Sequence model (Shibuya and Hovy 2020).

### 3.1 Models

MRC task is formulated in the following way: for the given context *X* and question *Q* the model should obtain answer *A* with some function *F* defined as A=F(X,Q). In the named entity recognition task, *X* would be the given sentence/paragraph; *Q* is some generated or selected query sentence for a given named entity type; *A* is the subsequence of the context *X* that denotes the named entity; *F* is the retrieving model itself.

For the MRC model, we employed three binary classifiers based on the output of the last hidden layer from the RuBERT model (Russian BERT) (Kuratov and Arkhipov 2019). The first classifier determines the starting position of the named entity. The second classifier determines the ending position of a named entity (possibly different) of the same class. The third classifier decides, whether chosen start-end pairs represent a single named entity of such class. These classifiers are trained for each class (type) separately. Batch size was set to 16 with maximum length of the sequence to be 192 tokens. Model was trained during 16 epochs on 8 T V100 GPUs. Other parameters are set to default values after (Li et al. 2020).

We compared several question variants for the MRC model.

**Keyword**: the question consists of a single entity tag such as DISO or ANATOMY (Li et al. 2020).

**Component-based**: 2–5–10 most frequent lemmatized components of a given entity are used for formulating a query, for example “DISO are entities such as a tumor, complication, disorder, disease, illness” (five-component example). Previous experiments with the general NEREL dataset showed that component-based questions outperformed other variants (Rozhkov and Loukachevitch 2022).

**Contextual**: a sentence from the training sample containing a named entity of a given type without explicit or implicit labeling used for this entity in the sentence. For example, a question for DISO entity type can be as follows: “60 patients in the most acute period of hemispheric ischemic stroke were examined.”

**Lexical**: as in the contextual variant, a sentence from the training corpus is used as a question; additionally, the entity of a given type is masked with its label (Zhou and Chen 2021). We used the so-called full lexical approach, when all entities in a sentence of a given type are substituted with masks. An example of a masking sentence with several mentions of an entity looks as follows. The initial sentence contains three mentions of DISO: “The addition of gout contributes to endothelial dysfunction and worsens the course of hypertension.” The corresponding lexical question is: “The addition of DISO contributes to DISO and worsens the course of DISO.” If a longer entity contains a shorter entity of the same type, the longer entity is preferred (so called outmost variant).

The selection of a sentence for contextual or lexical questions is carried out in the following manner:

- The most frequent entity for a given entity type is selected;
- The first sentence in the training set that contains the selected entity is extracted to be used as a question. By “first” we imply here the lexicographic order of the filenames of the original dataset.

We also provide experimental results for the *second-best Sequence model* (Shibuya and Hovy 2020) since it gave comparable results in the NEREL dataset. The model treats the tag sequence for nested entities as the second best path within the span of their parent entity. In addition, the decoding method for inference extracts entities iteratively from outermost ones to internal ones in an outside-to-inside way. It uses the Conditional Random Field method as an output layer. For this setup, we employed RuBERT model with batch size set to 16 and the same length of 192 tokens. The model was trained for 32 epochs on 8 GPUs while other parameters were set to default values.

### 3.2 Results

Span-level micro- and macro-averaged precision, recall, and F1 results of the models are shown in Table 7. The performance of the five-component MRC model for the 10 most frequent entities is presented in Table 8.

**Table 7:** Results of nested NER models on NEREL-BIO.

Model	Precision	Recall	MICRO-F	MACRO-F
MRC				
Keyword	76.95	75.80	76.36	58.42
2-comp	**77.86**	76.16	**77.00**	57.93
5-comp	77.25	76.26	76.74	57.27
10-comp	76.99	76.22	76.60	57.20
Lexical	77.1	75.91	76.75	**59.68**
Contextual	72.66	**76.94**	74.72	59.09
Second-best	75.28	72.98	74.10	51.29

Best results are marked in bold.

**Table 8:** Results of the lexical variant of the MRC model on most frequent entity types

Model	Precision	Recall	F1
ANATOMY	82.77	85.27	83.99
CHEM	80.74	81.94	81.32
DATE	74.85	78.43	76.59
DISO	79.83	82.29	81.03
LABPROC	74.13	60.28	66.47
MEDPROC	70.86	77.37	73.96
NUMBER	83.48	90.38	86.79
PERCENT	94.76	94.51	94.63
PERSON	85.30	93.80	89.35
PHYS	58.76	62.02	60.31

As shown in Table 7, the described variants of the MRC model (except the contextual variant) obtain comparable results in Micro-F measure. The best macro-averaged results are achieved by the lexical variant. Depending on entity type, performance of the MRC model varies greatly (see Table 8). In particular, this model achieves 85% F1 and 61% on ANATOMY and PHYS, respectively. We note that the best obtained results of nested NER for NEREL-BIO are lower than for general NEREL dataset, on which the MRC model achieved more than 80% micro-F-measure. This is in line with existing published NER results that also show similar decreased results on biomedical texts (Shibuya and Hovy 2020; Liu et al. 2022). The results for second-best sequence model are closest to the MRC model in micro measures but significantly worse in macro measures. This can be partly explained by the low amount of training data for specific entity types (Artemova et al. 2022).

### 3.3 Error analysis

We analyzed the results of the best MRC model on the NEREL-BIO test collection in comparison with manual annotation and found the following distribution of errors: (i) missed entities—33%; (ii) misclassification of entities—32%, including misclassification of abbreviations, which can be of different entity types but look very similar: IPN (iskrivliniye peregorodki nosa—deviated septum of the nose), MPT (methadone maintenance therapy); (iii) extra entities—27% including evidently longer entities than necessary: verb groups (“subgroup was taken in the 2nd group”), conjunctive groups (“ART and MMT”), etc., with a comma in the middle (“EMBASE, Medline”), etc. Also some irrelevant entities can be labeled, for example, “level of education” was classified as PHYS; (iv) different boundaries of the same entity −8%: “cohort studies” or “cohort studies of mortality”. Missing entities were found in human markup due to the difficulty of the annotation task itself. For example, in the “mild cognitive impairment” phrase, an annotator missed labeling “cognitive impairment”.

## 4 Discussion and limitations

Several issues may potentially limit the applicability of NEREL-BIO; they are mostly shared with other available datasets.

### 4.1 Seen and unseen mentions of entities

Recent works on BERT-based models for information extraction demonstrate that the generalization ability of these models is influenced by domain shift or whether the test entity/relation has been seen in the training set (Miftahutdinov et al. 2020; Tutubalina et al. 2020; Kim and Kang 2022). To avoid such biases, Kim and Kang (2022) removes overlaps in entity mentions and concept identifiers between training and test sets while Tutubalina et al. (2020) focuses on zero-shot entity linking between different concept terminologies. We leave these approaches to future work. We plan to investigate how well MRC models for nested NER can be adapted to unseen mentions.

### 4.2 Knowledge transfer between general and biomedical domains

The proposed NEREL-BIO corpus shared annotation scheme with our general-domain dataset NEREL for common entity types such as AGE, NUMBER, FACILITY, and ORGANIZATION (21 types in total). Transferability of trained models across two datasets with completely different contexts can be limited due to domain shift, while sequential training can cause complete retraining of model weights. We mark the investigation of strategies for combining different domains for future work.

### 4.3 Disease-centric abstracts

NEREL-BIO includes PubMed abstracts describing the results of clinical trials, hospitalization, and treatment of patients. The most frequent entities (e.g. diseases, injury, and anatomy) are related to a clinical domain, while biological entities such as genes and proteins are less presented. We suppose that this restricts the extraction of new biological relationships for protein-protein interaction or knowledge graph completion tasks, which will require additional data annotation.

## 5 Conclusion

Biomedical texts contain numerous nested mentions of entities such as anatomical parts within each other, diseases containing body parts or chemicals, names of procedures, which include diseases or devices, etc. In this article, we presented the first Russian dataset of biomedical abstracts NEREL-BIO, annotated with nested entities. The selected abstracts focus primarily on diseases and related medical procedures. The dataset contains a small collection of annotated parallel English abstracts. Our annotation shows that nested entities provide a better basis for extracting relations that would otherwise be lost. Similarly, nested entities also permit more complete entity linking to knowledge bases. Since, NEREL-BIO extends the annotation scheme of the general-domain Russian NEREL dataset, it permits studying domain transfer methods.
